# Developing and validating a questionnaire to assess the symptoms of blepharitis accompanied by dry eye disease

**DOI:** 10.1007/s00417-023-06104-2

**Published:** 2023-05-27

**Authors:** Haoyu Li, Daniel Böhringer, Philip Maier, Thomas Reinhard, Stefan J. Lang

**Affiliations:** Eye Center, University of Freiburg, Killianstr. 5, 79106 Freiburg, Germany

**Keywords:** Blepharitis, Dry eye disease, Ocular surface disease index

## Abstract

**Purpose:**

To propose additional items for established dry eye disease (DED) instruments that cover blepharitis-specific signs and symptoms and to determine the association between the clinical findings and subjective complaints.

**Methods:**

Thirty-one patients with blepharitis and DED were prospectively included in the pretest period for selecting suitable questions. In the main phase of the study, the selected questions were then tested on 68 patients with blepharitis and DED and 20 controls without blepharitis or DED. Pearson’s coefficient of correlation was calculated between the blepharitis-specific questions, tear break-up time (TBUT), the Schirmer test score, and the ocular surface disease index (OSDI) score; and the similarity between the blepharitis-specific questions, OSDI questions, and objective parameters for DED was assessed via hierarchical clustering. Furthermore, the discriminatory power of the blepharitis-specific questions was investigated with the receiver operating characteristic (ROC) curve.

**Results:**

The additional question about heavy eyelids revealed a significant correlation with the OSDI score (*r* = 0.45, *p* < 0.001) and Schirmer score (*r* =  − 0.32, *p* = 0.006). Cluster analysis demonstrated the similarity between the question about heavy eyelids and TBUT. In addition, the OSDI questionnaire had the highest discriminatory power in ROC analysis, and the OSDI score significantly correlated with the specific questions about eyelids sticking together (*r* = 0.47, *p* < 0.0001) and watery or teary eyes (*r* = 0.34, *p* = 0.003).

**Conclusions:**

The blepharitis-specific additional questions were closely associated with objective parameters for DED. The question about heavy eyelids might be well suited for recording the symptoms of hyposecretory and hyperevaporative dry eye with blepharitis.
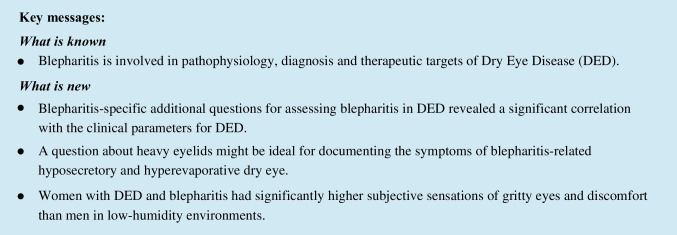

## Introduction

The fundamental mechanism of dry eye disease (DED) includes tear hyperosmolarity resulting from reduced lacrimal secretion and/or a tear film lipid layer deficiency related to meibomian gland dysfunction (MGD). In MGD, the formation of lipases and esterases through proliferation of Staphylococcus, *Propionibacterium acnes*, *Bacillus oleronius*, and Demodex may cause changes in polar lipid composition and increased melting temperature in meibum, which can lead to hyperevaporative dry eye [1, 2]. Conversely, DED can give rise to the release of inflammatory mediators and proteases, a tear film instability, and meibomian gland orifice keratinization [3].

Chronic blepharitis may affect 37–47% of all patients who visit an ophthalmologist [4]. Keratoconjunctivitis sicca was found in 25–50% of patients with chronic blepharitis [1]. Seventy-eight percent of dry eye sufferers, on the other hand, had chronic blepharitis [5]. The symptoms of chronic blepharitis are usually due to its association with keratoconjunctivitis sicca and can be listed as follows: burning, irritation, tearing, redness, crusting and sticking of the eyelids, as well as visual issues such as photophobia and blurred vision.

The use of a questionnaire, such as the ocular surface disease index (OSDI) or Standard Patient Evaluation of Eye Dryness (SPEED), can help uncover or track symptoms related to ocular discomfort in DED patients. The OSDI has become established for wide use in clinical trials with a good specificity of 83% and a moderate sensitivity of 60%, composed of 12 questions about the frequency of DED symptoms, their impact on quality of life, and possible environmental triggers [6]. Each OSDI question is rated on a scale of 0 to 4: (0) none of the time, (1) some of the time, (2) half of the time, (3) most of the time, and (4) all of the time. The overall OSDI score is computed using the formula: OSDI = [(sum of scores for all questions answered) × 100] / [(total number of questions answered) × 4]. The more severe the symptoms, the higher the OSDI score. Although significant Spearman’s rank correlation between the OSDI and blepharitis-associated questions has been identified, a detailed overview of the association between blepharitis-specific symptoms and clinical parameters for DED is lacking [7].

This study aimed to suggest additional items for established DED questionnaires that quantify blepharitis-specific signs and symptoms, to correlate the blepharitis-specific questions with objective parameters for DED, and to provide new insights into the association between the clinical findings and subjective complaints in DED and blepharitis. Patient’s symptoms might be quantified with the help of blepharitis-specific questions, making it possible to accurately assess the efficacy of a therapy and establish therapy monitoring.

## Methods

### Patients

In order to pick appropriate questions in the main phase of the study, we prospectively enrolled 31 patients with concurrent blepharitis and DED who had answered 43 different blepharitis-associated questions at the Eye Center of Freiburg University between 2019 and 2020 in the pretest phase. The seven selected questions from the pretest phase were then assessed on 68 patients with blepharitis and DED in the study’s main phase. Blepharitis, characterized by inflammation of the eyelids, can be diagnosed by symptoms and signs, e.g., redness, irritation, burning, tearing, itching, crusting of eyelashes, loss of eyelashes, eyelid sticking, blurring or fluctuating vision and photophobia along with the measurement of visual acuity, an external examination of skin and eyelids, slit-lamp biomicroscopy including the evaluation of eyelid margin, eyelashes, conjunctiva, cornea, tear meniscus, tear film break-up time, and pattern. Twenty patients’ companions without blepharitis or DED were employed as healthy controls free of eye diseases to test the specificity of additional questions, who might have similar environmental factors and family history to the patients. Clinical data, such as age at diagnosis, sex, vision, slit lamp exam results, tear break up time (TBUT), Schirmer test score with anesthesia, questionnaire responses and photo documentation, were collected. The exclusion criteria were ocular surface diseases other than DED, e.g., atopic keratoconjunctivitis, herpetic keratoconjunctivitis, ocular pemphigoid, and ocular graft-versus-host disease (GVHD). This study was approved by the Ethics Committee at the University of Freiburg (approval number Nr. 301/19). An anonymous analysis was conducted in accordance with local ethical guidelines.

### Questionnaire design and clinical tests

In the pretest phase, patients were asked about the intensity with a visual analogue scale (VAS) and the frequency of the following symptoms: gritty eyes, dry eyes, itchy eyes, heavy lids, eye/eyelid burning, eyes sensitive to light, tired eyes, blurred vision, watery or teary eyes, crusting/flakes on the eyelids, eyelids sticking together (especially in the morning), swollen lid margins, loss of eyelashes, and eyelid redness. Patients needed to answer how often the symptom occurred during the last week by selecting the appropriate option from “All of the time,” “Most of the time,” “Half of the time,” “Some of the time,” and “None of the time.” Additionally, questions about the limitations of reading, driving at night, using the computer, watching TV, sleeping, using a mobile phone, and wearing contact lenses were included. Finally, patients were asked if they were generally most bothered by how their eyes look or feel.

**Fig. 1:**
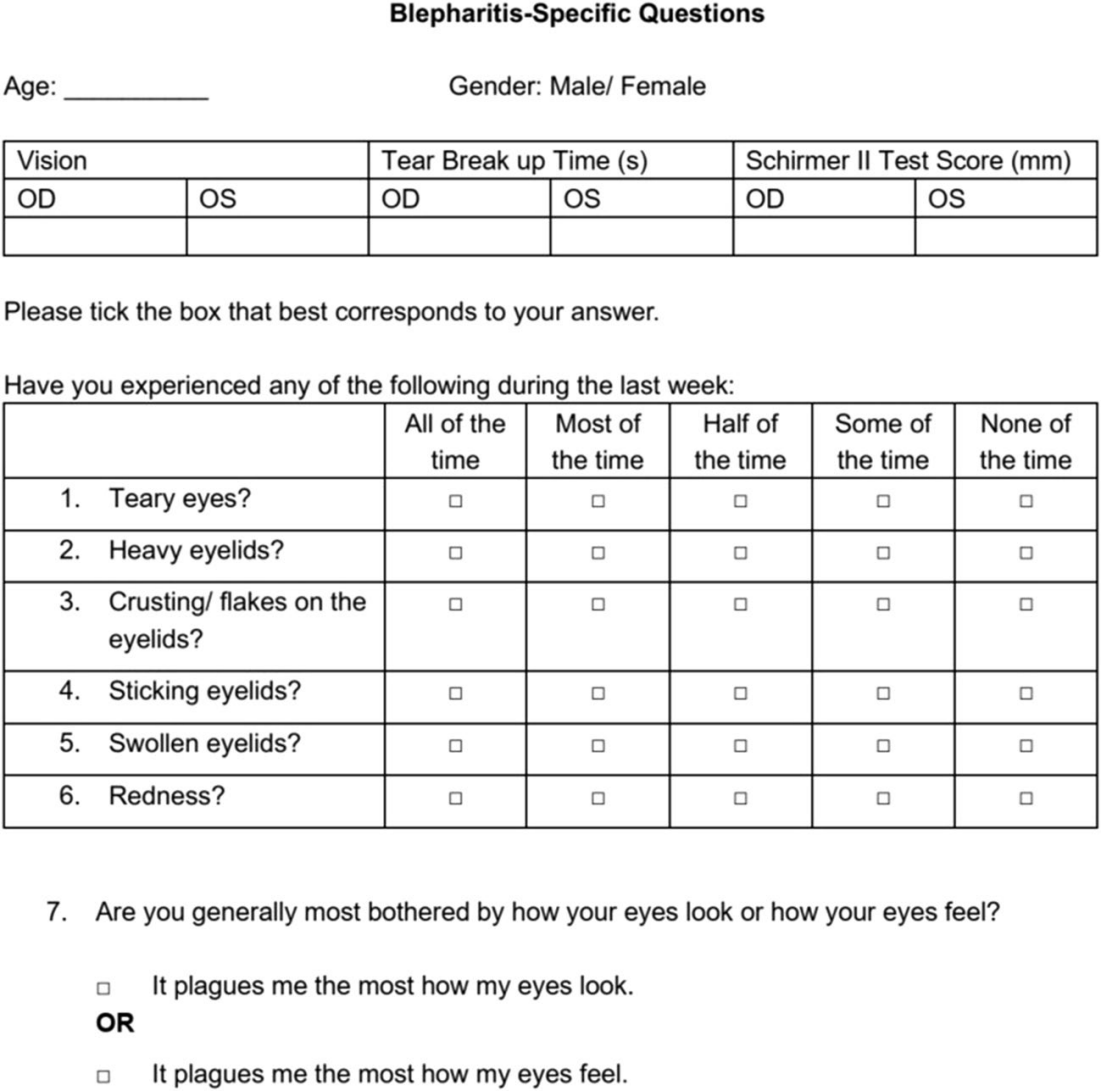
Blepharitis-specific questions in the main phase of the study

By analyzing the correlation of the questions and the distribution of the answers, the questions about teary eyes, heavy eyelids, crusts/dandruff on the eyelids/eyelashes, sticking eyelids, swollen eyelid margins, and red eyelid margins were selected as the appropriate additional question in the main phase (Fig. 1). The question, if they were generally most bothered by how their eyes look or feel, was also chosen as representative of the patient’s subjective feelings. In addition, the OSDI was used in the main phase to compare the validity between the OSDI and the blepharitis-specific additional questions. This clinical study has obtained copyright permission for the German version of the OSDI.

The most widely used test of tear film stability in clinical practice has been the TBUT test [8]. A strip of fluorescein (FLUO® STRIPS Contacare Ophthalmics and Diagnostics) was moistened with saline (NaCl 0.9% Fresenius) and stained the lower fornix. The time from the last blink to the first rupture of the tear film with visible black spots was measured and documented. The Schirmer II test was used in this study to determine baseline lacrimal secretion. After 5 min of topical anesthesia with oxybuprocaine eye drops (Novesine® 0.4% OmniVision GmbH), a filter paper strip (Schirmer Tear Test® HS Clement Clarke Ophthalmic) was bent at the rounded end and hooked into the saccus conjunctivae at the border of the temporal to the medial third of the lower lid. The length of filter paper soaked after 5 min was recorded as the Schirmer test score. There has been a reported range of sensitivity (77–85%) and specificity (70–83%) values [9, 10].

### Statistical analysis

To select appropriate questions for the main phase of the study, correlation heat map of 43 blepharitis-associated questions and clinical parameters in the pretest phase were generated, and distribution of answers to all questions was compared. For descriptive statistical analysis, median values for age at diagnosis, vision, TBUT, the Schirmer test score, and the OSDI score recorded in the study’s main phase were calculated. To examine association between the clinical findings and subjective complaints, Pearson’s coefficient of correlation between the blepharitis-specific questions and clinical parameters for DED was calculated, followed by hierarchical clustering and an analysis method using a generalized linear model. Unless otherwise stated, the ophthalmological parameters of the right eye were selected for quantitative analysis in the main phase because there was a strong positive correlation between the clinical data of the right and left eye. Furthermore, we compared the discriminatory power of the blepharitis-specific questions and the OSDI with the receiver operating characteristic (ROC) curve. Moreover, patients were divided into two groups according to sex, and the sex-specific differences in symptoms were investigated by comparing the distribution of answers. All calculations were performed using the R-System (available at: ww.r-project.org, V4.1.2). A significance level was set at 0.05. We did not adjust for multiple testing due to the explanatory character of our investigation.

## Results

### Patient characteristics

Sixty-eight patients with blepharitis and DED and 20 controls without blepharitis or DED were included in the main phase of the study. The characteristics of the patients and the control group are summarized in Table 1. The median age of the patient group was 69 years (interquartile range (IQR) 24.5 years), composed almost equally of 33 men (49%) and 35 women (51%). The individual OSDI score of all patients included in the main phase had a median value of 32.9 (IQR 33.8). The median clinical parameters for the left and right eyes were comparable. The distribution of TBUT, the Schirmer test score, and the OSDI score showed that the data covered patients with different severities of DED very well [11].

**Table 1:** Characteristics of the patients and the control group

Group	Patients (*n* = 68)	Controls (*n* = 20)
Age in years, median (Q1, Q3)	69.0 (53.8, 78.3)	57.0 (48.5, 67.0)
Vision OD, median (Q1, Q3)	0.8 (0.6, 1.0)	1.0 (0.8, 1.0)
Vision OS, median (Q1, Q3)	0.8 (0.6, 1.0)	1.0 (0.95, 1.0)
TBUT (OD) in s, median (Q1, Q3)	3.2 (2.1, 5.5)	10.0 (10.0, 10.0)
TBUT (OS) in s, median (Q1, Q3)	3.0 (1.8, 5.3)	10.0 (10.0, 10.0)
Schirmer test score (OD) in mm, median (Q1, Q3)	5.0 (2.0, 10.0)	19.0 (14.5, 20.0)
Schirmer test score (OS) in mm, median (Q1, Q3)	5.5 (2.0, 10.0)	18.0 (14.0, 20.0)
OSDI score, median (Q1, Q3)	32.9 (20.2, 54.0)	4.1 (0, 6.2)

### The frequency of heavy eyelids was associated with hyposecretory and hyperevaporative dry eye

**Fig. 2:**
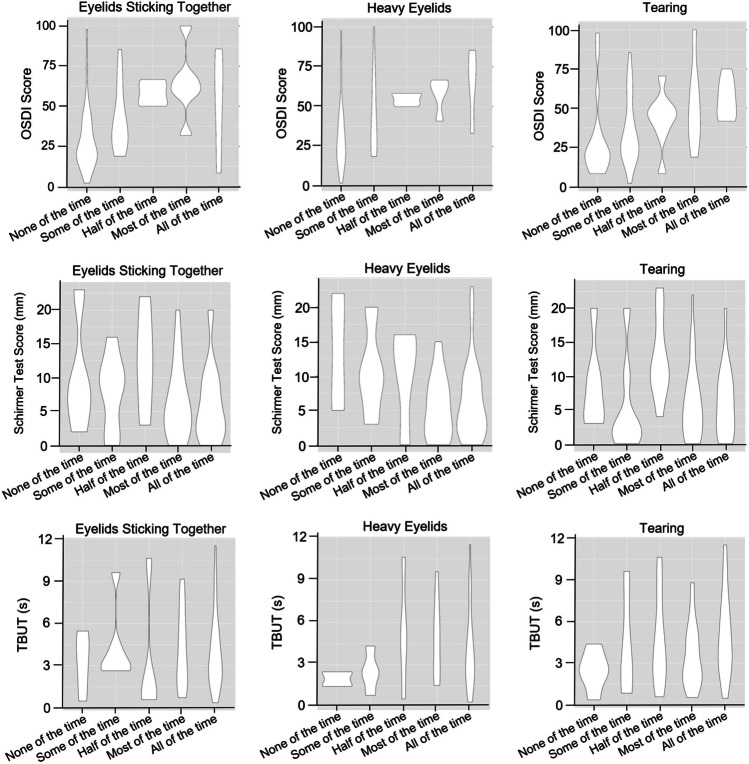
Violin plot visualization of the bivariate correlation between blepharitis-specific additional questions about eyelids sticking together, heavy eyelids and tearing, and clinical parameters for DED

A bivariate correlation analysis of three blepharitis-specific additional questions about eyelids sticking together, heavy eyelids, and tearing, the OSDI score, TBUT, as well as the Schirmer test score with violin plot visualization (Fig. 2) were performed, and the Pearson’s correlation coefficient and *p* value were determined and demonstrated in Table 2. The OSDI score correlated significantly with the questions about eyelids sticking together (*r* = 0.47, *p* < 0.0001), heavy eyelids (*r* = 0.45, *p* < 0.001), and tearing (*r* = 0.34, *p* = 0.003). In addition, a significant negative correlation was found between the Schirmer test score and heavy eyelids (*r* =  − 0.32, *p* = 0.006), indicating a possible association between heavy eyelids and hyposecretory dry eye. However, no significant correlation was observed between TBUT and the blepharitis-specific additional questions.

**Table 2:** Pearson’s coefficient of correlation between blepharitis-specific additional questions and clinical parameters for DED

Objective parameter	Eyelids sticking together	Heavy eyelids	Tearing
OSDI score	*r* = 0.47 *p* < 0.0001	*r* = 0.45 *p* < 0.001	*r* = 0.34 *p* = 0.003
Schirmer test score (OD)	*r* = − 0.20 *p* = 0.09	*r* = − 0.32 *p* = 0.006	*r* = − 0.12 *p* = 0.30
TBUT (OD)	*r* = 0.03 *p* = 0.77	*r* = 0.14 *p* = 0.25	*r* = 0.16 *p* = 0.18

**Fig. 3:**
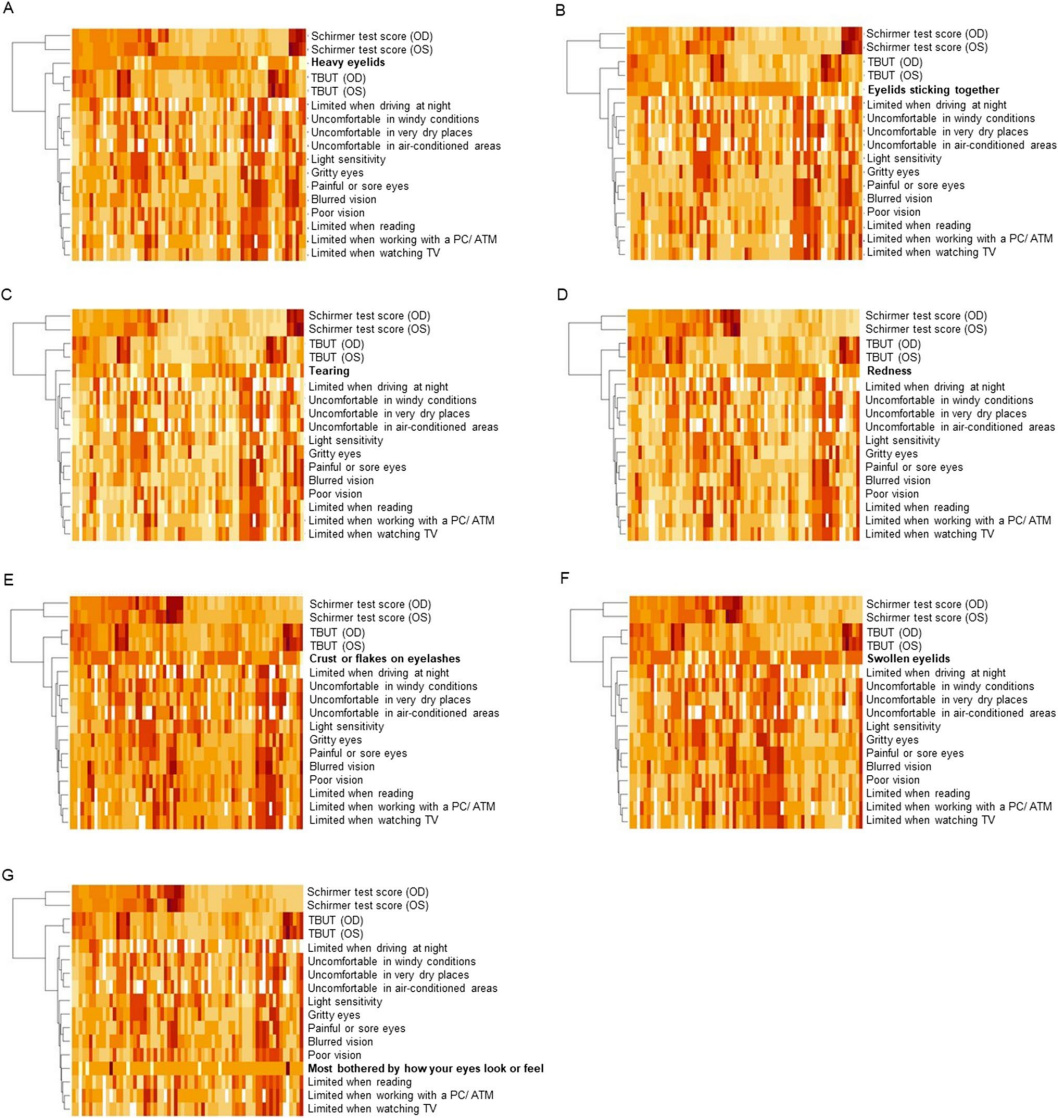
Cluster analysis of the seven blepharitis-specific additional questions in the main phase, the OSDI questions, TBUT, and the Schirmer test score. Each column represents a patient. Each row represents one blepharitis-specific additional question. Similarity structures were detected for the questions about heavy eyelids **a**, eyelids sticking together **b**, tearing **c**, redness at the eyelid margins **d**, crust or flakes on eyelashes **e**, swollen eyelid margins **f,** and the question whether the patient is more bothered by how the eyes look or the eyes feel **g**

Cluster analysis visualized with dendrogram revealed similarity structures for all blepharitis-specific additional questions in the main phase, the OSDI questions, TBUT, and the Schirmer test score (Fig. 3). Figure 3 a showed that the question about heavy eyelids and TBUT belonged to the same cluster, while all of the OSDI questions were in a self-contained group, implying that the question about heavy eyelids was closer to TBUT than the OSDI according to the distance or similarity measures used. Further grouping of all remaining OSDI questions showed that the questions about poor vision and limitations in reading, computer work and watching TV, as well as the question about whether the patient is more bothered by how the eyes look or feel, belonged to a cluster at a different level (Fig. 3g), illustrating the association between subjective sensation and quality of life in relation to vision.

### The OSDI score correlated significantly with blepharitis-specific additional questions

**Fig. 4:**
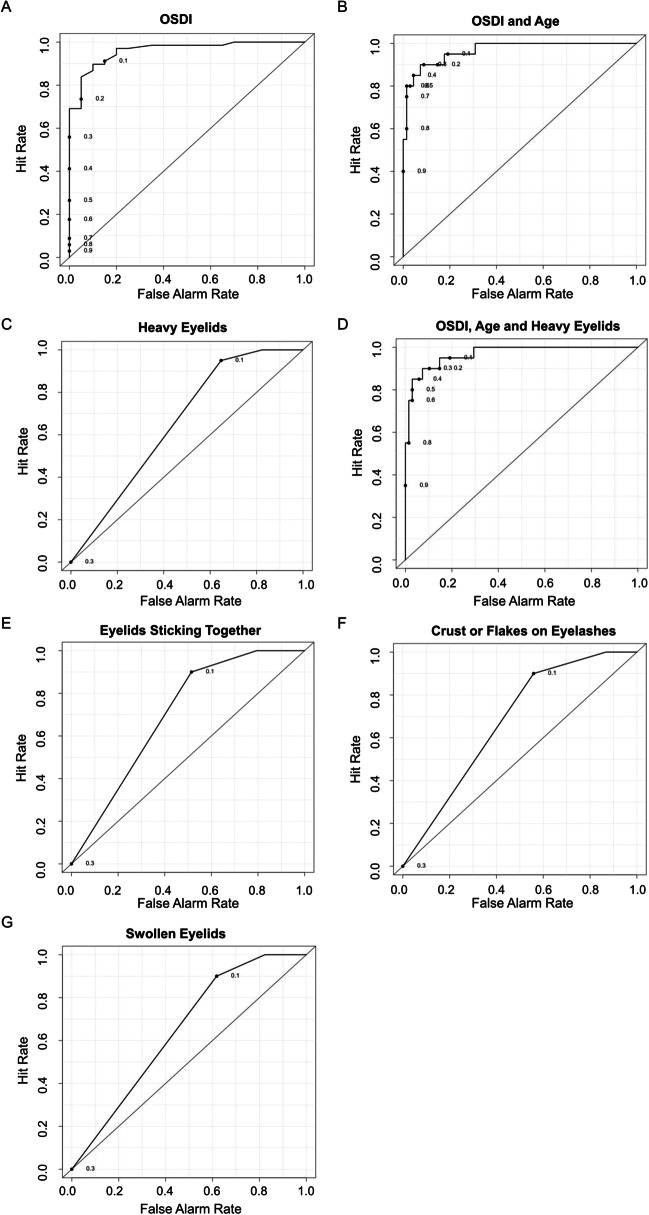
ROC curves for the discriminatory power analysis of the OSDI and blepharitis-specific additional questions in the main phase of the study

In the ROC analysis, the “hit rate” referred to the proportion of the patient group identified by the question in the questionnaire, i.e., the percentage of patients in the patient group that had the symptom. The “false alarm rate” described the percentage of the control group that the questionnaire question detected, namely, the proportion of the controls with the symptom in the control group. The 12 OSDI questions had the highest discriminatory power between the controls without DED or blepharitis and the patients with DED and blepharitis, outperforming the additional questions about eyelids sticking together, heavy eyelids, crust or flakes on eyelashes, and swollen eyelid margins (Fig. 4). Moreover, the discriminatory power was further improved by the combination of the OSDI, the question about heavy eyelids, and age at diagnosis compared to the OSDI alone.

A generalized linear model was used to test whether the results of all blepharitis-specific additional questions in the main phase could be predicted by the OSDI score, the Schirmer test score, and TBUT. The test results revealed that the OSDI score was a significant factor in the models for the questions about eyelids sticking together (coefficient = 0.044, *p* = 0.006), tearing (coefficient = 0.032, *p* = 0.018), heavy eyelids (coefficient = 0.049, *p* = 0.024), redness at the eyelid margins (coefficient = 0.029, *p* = 0.038), and crust or flakes on eyelashes (coefficient = 0.032, *p* = 0.046), demonstrating that the OSDI score significantly correlated with these blepharitis-specific additional questions. Additionally, the Schirmer test score was a significant factor in the models for the questions about heavy eyelids (coefficient = 0.14, *p* = 0.027) and swollen eyelid margins (coefficient = 0.12, *p* = 0.03).

### Women with DED are more likely than men to experience symptoms

Significant sex differences in the distribution of answers were compared. Thirty-one percent of female patients answered “Most of the time” to the OSDI question about discomfort in places with low humidity, whereas 11% of male patients gave the same answer (*p* = 0.015). In addition, 20% of women chose the option “All of the time” for the OSDI question about gritty eyes, while no men responded with the same answer (*p* = 0.039). Overall, these results indicated that women had stronger subjective perceptions of gritty eyes and feeling uncomfortable in very dry places than men.

## Discussion

Blepharitis with structurally and functionally inadequate meibomian glands plays an important role in the pathophysiology of DED. The signs and symptoms of blepharitis represent the microbial growth, an inflammation of the eyelid, the conjunctiva and the cornea, as well as tear film instability [12].

This study demonstrates that the blepharitis-specific additional questions can assess the symptoms of patients with blepharitis and DED, when significantly correlated with the clinical parameters for DED. Our results are in line with a psychometric study presenting a significant strong correlation between the OSDI and blepharitis-associated symptoms, which included heavy or swollen eyelids, redness, watery eyes, crusty or scaly eyelids, and eyelids sticking together [7]. Among the seven blepharitis-specific additional questions in the main phase of the study, the question about heavy eyelids was the most appropriate for capturing the symptoms of blepharitis and DED, due to its significant correlation with the OSDI score (*r* = 0.45, *p* < 0.001) and the Schirmer test score (*r* =  − 0.32, *p* = 0.006) and the same cluster membership as that of TBUT in cluster analysis. The similarity between the question about heavy eyelid and TBUT in cluster analysis may be explained by the fact that meibomian gland dysfunction can lead to the tear film instability, increased evaporation and eventually a hyperevaporative dry eye. Taken together, we concluded that the question about heavy eyelids could be used to assess symptoms of both hyposecretory dry eye and hyperevaporative dry eye, while the OSDI was more applicable to deficiency of the aqueous-mucinous tear film components due to lack of correlation with the parameters for diagnosing hyperevaporative dry eye [13]. The coefficient of determination is 0.20 with a Pearson’s correlation coefficient of 0.45. The deviance residuals were Min 1Q Median 3Q Max − 1.76134 − 0.35119 − 0.17466 − 0.09453 1.96727. The reproducibility, relative validity, and responsiveness of the additional questions including the question about heavy eyelids are needed to demonstrate through follow-up studies for a potential application of appropriate questions in routine clinical practice.

Furthermore, a significant sex-specific difference was determined in the distribution of answers to the OSDI questions about discomfort in places with low humidity (*p* = 0.015) and gritty eyes (*p* = 0.039). This observation is in accord with another study suggesting that women with DED are more likely than men to experience symptoms such as feeling uncomfortable with environmental triggers [14]. There are significant sex-specific differences in morphological appearance and gene expression of the cornea, the lacrimal gland, the meibomian gland, and the tear film, which may play a role in the development of DED [15].

The frequent coexistence of DED and blepharitis allowed for the prospective character of the current study with good data quality and high power. Despite the limited number of study participants, we detected a significant correlation between the blepharitis-specific additional questions and objective clinical parameters. However, no significant correlation between TBUT and the blepharitis-specific additional questions was noted in this study. Finis et al. [13] also found no significant correlation between TBUT and the OSDI score, but a significant negative correlation between the SPEED (Standardized Patient Evaluation of Eye Dryness) questionnaire and lipid layer thickness of the tear film, which indicated hyperevaporative tear deficiency. Unlü et al. [11] successfully determined a significant negative correlation between TBUT and the OSDI (*r* =  − 0.38, *p* = 0.02) with a specific collective of computer users. The discrepancy between clinical signs and symptoms in DED is consistent with the notion that sensitization of the ocular sensory pathways may be triggered by events occurring long before the patient visits the ophthalmic clinic [16–19]. When peripheral nerve injury or inflammation causes functional and structural changes in the ocular pain pathways due to disturbed ocular surface homeostasis, central pain may develop and persist regardless of peripheral nociceptive input [20].

In the ROC analysis, the OSDI score (or the 12 OSDI questions?) had the highest discriminatory power in distinguishing the control group from the patient group, outshining any blepharitis-specific additional questions. This may be attributed to the fact that most of the controls in the main phase of the study did not suffer from DED, so that the blepharitis-specific additional questions could not show any difference. Future studies could attempt to include a group of patients with DED and without blepharitis to test the discriminatory power of the blepharitis-specific additional questions. The findings might be impacted by age and sex disparities between the patients and healthy controls, as old age and female sex are risk factors linked to DED.

In conclusion, the present study exhibits significant correlations between the clinical parameters for DED and the blepharitis-specific additional questions for assessing blepharitis in DED. The question about heavy eyelids may be best suited to describe the symptoms of hyposecretory and hyperevaporative dry eye with blepharitis due to significant correlations with the OSDI score and the Schirmer test score, as well as the same cluster membership as from TBUT in cluster analysis. In addition, this study demonstrated that women with DED and blepharitis had significantly stronger subjective perceptions of gritty eyes and discomfort than men in places with low humidity.

There are limitations to this study. With a small study pool, caution must be applied, as the results might be lack of reliability due to insufficient range of responses. It is necessary to prove the specificity of the additional questions for blepharitis with quantitative measurements of meibomian gland dysfunction such as lipid layer thickness by LipiView interferometer. As fluorescein makes the tear film less stable, the measurement of TBUT may not be an accurate representation of its condition. Spearman’s correlation coefficient and internal consistency reliability, often measured using Cronbach’s alpha, should be calculated for the values with non-normal distributions.

The significant correlations between the objective parameters for dry eye disease und the questions about signs and symptoms of blepharitis reflected the close relationship between dry eye disease and blepharitis. Case–control studies, prospective cohort studies, molecular genetic analysis, and bioinformatics analysis will be required to investigate the relationship between dry eye disease and blepharitis.
